# Examination of Structural Variations of the Circle of Willis by 3D Time-of-Flight Magnetic Resonance Angiography

**DOI:** 10.3389/fnins.2020.00071

**Published:** 2020-02-11

**Authors:** Jing Li, Jue Wang, Xiao-er Wei, Yu-wu Zhao, Feng Wang, Yue-hua Li

**Affiliations:** ^1^Shanghai Jiao Tong University Affiliated Sixth People's Hospital, Shanghai, China; ^2^Shanghai Tenth People's Hospital, Tongji University, Shanghai, China

**Keywords:** magnetic resonance angiography, digital substraction angiography, brain vascular malformation, circle of Willis, 3D-TOF-MRA

## Abstract

**Objectives:** To explore structural variations of the circle of Willis using three-dimensional time-of-flight magnetic resonance angiography (3D-TOF-MRA), and to compare this modality with digital subtraction angiography (DSA).

**Methods:** A total of 819 consecutive patients suspected of having cerebral vascular diseases underwent 3D-TOF-MRA, followed by DSA within 2 weeks. We report accuracy, sensitivity, specificity, positive predictive value (PPV), and negative predictive value (NPV) of 3D-TOF-MRA compared with DSA.

**Results:** The sensitivity and specificity of combined analyses were 90–100 and 98–100%, respectively. The sensitivity and NPV of 3D-TOF-MRA images for A-, C-, D-, and H-types of circle of Willis anomalies were 100%. The specificity, accuracy and sensitivity were all 100% for detecting absence of the anterior communicating artery (ACOA). Sensitivity, specificity, PPV, and NPV were all 100% for detecting F-type. The sensitivity and PPV of volume rendered (VR) images for the B-, E-, and G-types were relatively low (85.0, 86.2, and 73.8%, respectively). Maximum intensity projection (MIP) was somewhat better (88.3, 89.2, and 81.8%, respectively). Combined analyses were better still (95.8, 96.1, and 99.0%, respectively). Specificity and NPVs were high (99.3–100%).

**Conclusions:** 3D-TOF-MRA compares well to DSA for evaluation of the structure of the circle of Willis. As 3D-TOF-MRA is a non-invasive modality, it may be preferred as a means to evaluate structural variations of the circle of Willis.

## Introduction

The circle of Willis is the most important intracranial pathway for collateral circulation. It plays critical roles in maintenance of stable intracranial blood flow and perfusion pressure. Developmental type, variation, and degree of opening of the structure all affect its function as a source of compensatory flow. Some studies suggest that aneurysm, stroke, and other cerebrovascular diseases are associated with structural variations of the circle of Willis (Alpers et al., 1959; Kayembe et al., 1984; Schomer et al., 1994). Therefore, it is important to conduct a large-scale epidemiological investigation so as to understand these variations, to explore the mechanism of cerebral vascular diseases, and to improve the accuracy of diagnosis and safety of treatment.

Current imaging methods include the transcranial Doppler (TCD), digital subtraction angiography (DSA), computed tomography angiography (CTA) and magnetic resonance angiography (MRA), of which DSA is the gold standard (Grolimund et al., 1987; Alberico et al., 1995; Katz et al., 1995). However, DSA is an invasive modality, with a number of drawbacks, such as radiation exposure, and requirement for contrast agent, and so forth. CTA is an excellent diagnostic modality, however, it also requires injection of contrast agent and exposure to radiation, with attendant effects on bone and calcification. Time-of-flight magnetic resonance angiography (3D-TOF-MRA), by contrast, is non-invasive, relatively inexpensive and efficient. There is no exposure to radiation or contrast agent. It can be applied to a wide number of indications (Krabbehartkamp et al., 1998). 3D-TOF-MRA has already been widely applied in diagnosis of cerebral vascular disease (Yano et al., 1997), however there is no large study comparing 3D-TOF-MRA with DSA, the gold standard for diagnosis. With the development of high-field-strength MR imaging, it is now possible to precisely and non-invasively visualize the microvasculature of the human brain, *in vivo*, using 3D-TOF-MRA (Hendrikse et al., 2008b; Vuillier et al., 2008; Chen et al., 2011).

The purpose of this study was to explore the structure of the circle of Willis using MR Angiography, and to compare the results to those of DSA as the standard.

## Materials and Methods

### Study Subjects

Between June 2007 and June 2016, 819 consecutive patients who were suspected cerebral vascular diseases detected by MRA were retrospectively collected. Before each patient was admitted to the hospital for an examination, an informed consent was signed, which agreed that all examination information will be used by the hospital in the research. They underwent DSA examinations within 2 weeks after MRA. We regarded the results of volume rendering (VR) DSA as the gold standard. To identify the components of the circle of Willis, we defined the A1 segment of the anterior cerebral artery (A1), the anterior communicating artery (ACOA), the P1 segment of the posterior cerebral artery (P1), and the posterior communicating artery (PCOA) from the results of DSA imaging.

**Figure 1:**
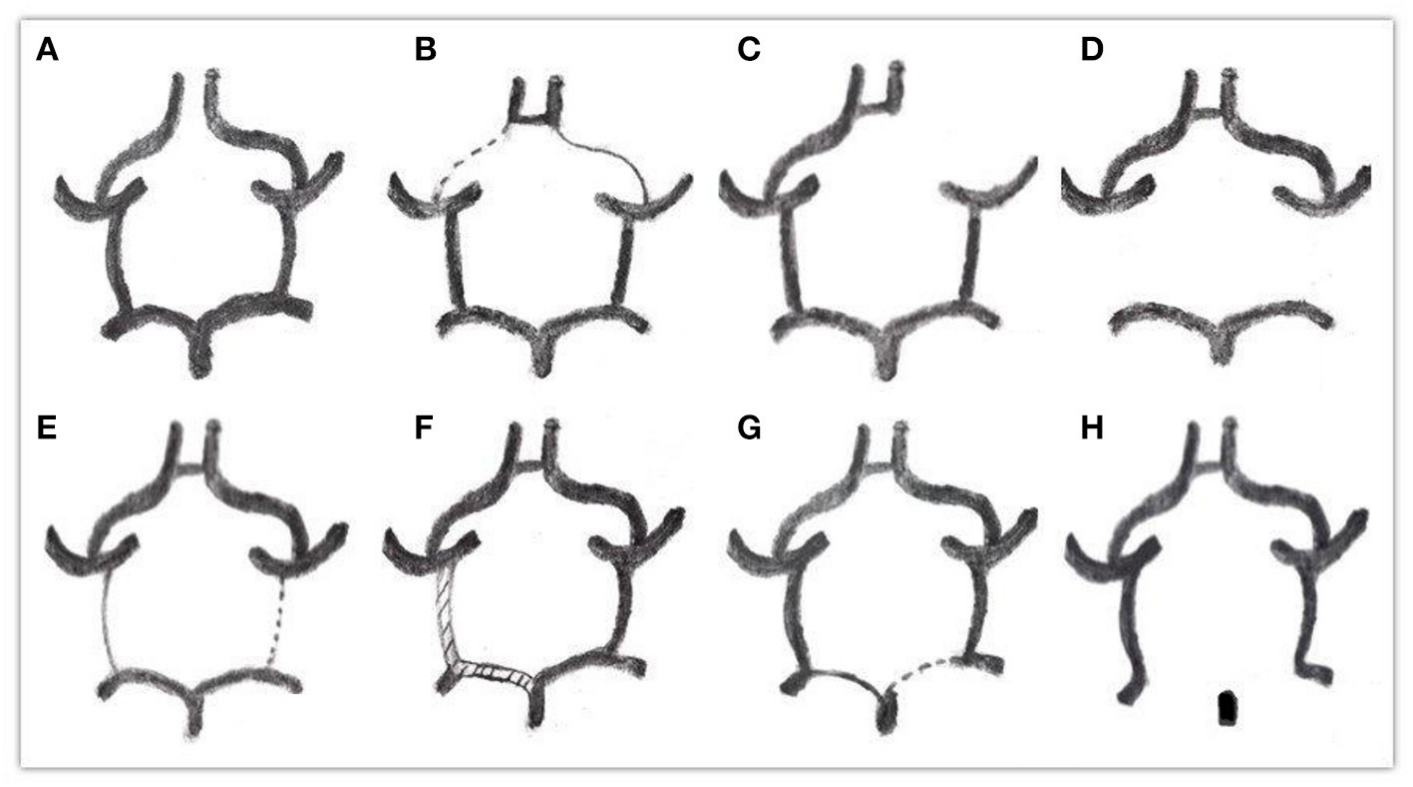
Classification of the circle of wills. **(A–H)** types.

Through observation and measurement of the ACOA, A1, PCOA, and P1 segment, the specific classification of types was as follows (Alpers et al., 1959; Riggs and Rupp, 1963) (Figure 1).

A: ACoA absent

B: One or both A1 arteries diameters below 1 mm

C: One or both A1 arteries absent

D: One or both PCOA arteries absent

E: One or both PCOA arteries diameters below 1 mm

F: One or both PCOA arteries diameters equal to P1's, all above 1 mm

G: One or both P1 arteries diameters below 1 mm, far less than PCOA

H: One or both P1 arteries absent.

### Image Acquisition

We perfomed all MRA examinations on a 3.0 T system (Achieva X-Series, Philips Medical Systems) with a Sense-Head-8 receiver head coil. 3D-TOF MRA was obtained using 3D T1-weighted fast field sequences with the following settings: field of view (FOV), 250 × 190 × 108 mm; voxel size, 0.342 × 0.7 × 0.6 mm; repetition time/echo time, 35/7 msec; four slabs (180 slices), slice thickness, 0.8 mm; flip angle, 20°; matrix, 732 × 1,024; acquisition time of 8 min and 56 s. Several techniques were used to ensure perfect image quality for visualizing small cerebral arteries, including SENSE (SENSitivity Encoding), MOTSA (multiple over-lapping thin-slab acquisitio), TONE (tilted optimized non-saturating excitation), and SLINKY (Sliding interleaved KY). Then we transferred the acquired image data sets to a workstation (EWS, Philips Medical). The 3D image reconstruction was performed by MIP (maximum intensity projection) and VR (Volume Rendering) with a specialized software package for 3D volume inspection with a 1,024 × 1,024 matrix (Philips Medical).

### DSA

An interventional neuroradiologist performed DSA within 14 days of MRA (median, 10.3 days; range, 1 h−14 days). We perfomed conventional 2D-DSA on a monoplanar unit (Axiom Artis VB22N, Siemens) with a 17–20 cm field of view (FOV) and a 1,024 × 1,024 matrix. We performed rotational angiography with 200° rotational run, an 8-s, injecting 3–4 mL contrast medium per second, and acquiring 200 images. We reconstructed the 3D images by VR on a workstation (SyngoXWP VA70B; Siemens) with a 1,283 × 5,123 matrix. Two observers (M.H.L. And W.W., with 22 and 15 years of experience, respectively, in neurointerventional radiology), who were blinded to previous imaging results and all clinical, identified the morphology of vessels in this study.

### Review of MRA Images

Two observers (Y. H. L., and X. E. W., with 18 and 15 years of experience, respectively, in neurointerventional radiology) were blinded to VR-DSA results and all clinical. They independently analyzed the 3D-TOF-MRA data sets on an offline-workstation with the use of the single artery highlighting approach from multiple on-screen viewing angles. In order to locate small vessels, the source images and MIPs were presented on-screen, thus allowing for adjusting the appropriate threshold of window width and level. The measurement of vascular diameter had a very important relationship with window width and window level. The W/L setting value of VR is constant, which is 450 ± 50/1,000 ± 50. The value of MIP is 1,300 ± 50/700 ± 50, and the value of original axial images is 1,000 ± 50/450 ± 50. With such parameters the vascular wall margin is very clear and the contrast is the highest.

We sought the starting point and end point of every vessel in the circle of Willis from the original images by slice tracking to estimate the continuity of vessels. Finally, three types of image were combined to the make an assessment. If a vessel in a VR and MIP image was not detected, or start/stop bits were not checked on the original images, a determination of absence of blood vessels was made. Discrepant conclusions were resolved by a third reviewer (M.H.L, with 20 years of experience in neurointerventional radiology). The diagnosis of the two observers was subjected to Kappa consistency assessment.

### Statistical Analyses

SPSS (version 13.0, SPSS Inc., Chicago, IL) was used for statistical analyses. DSA was regarded as the standard for analyzing differences in specificity, sensitivity, positive predictive value (PPV), negative predictive value (NPV), and accuracy between the original images, VR images, MIP images, and combined analyses.

## Results

**Figure 2:**
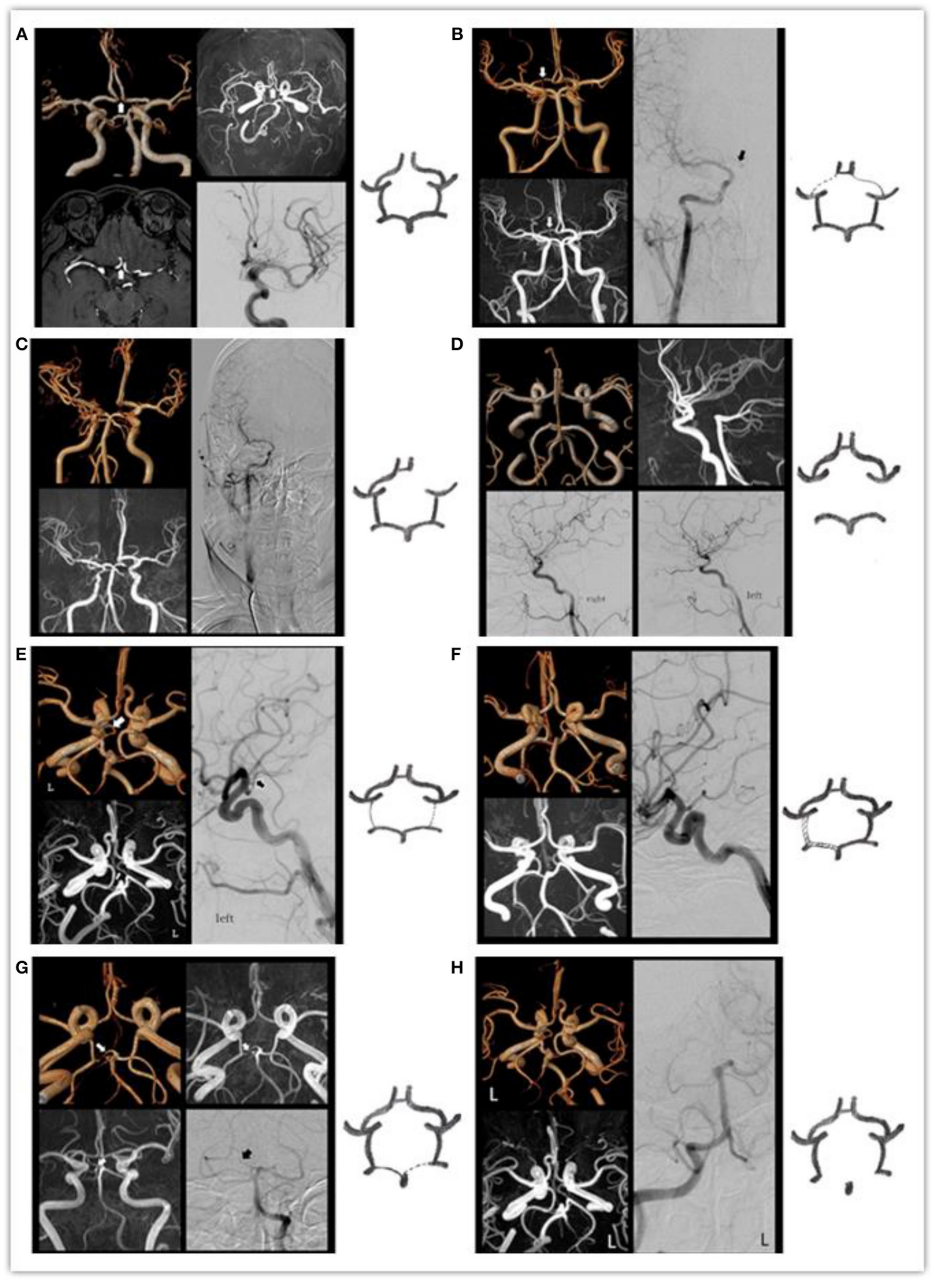
The MRA and DSA of the various types of the circle of Willis: **(A)** arrow indicates absent ACOA. **(B)** Arrow indicates right slim A1. **(C)** Absent right A1. **(D)** Absent bilateral PCOAs. **(E)** Arrow indicates left slim PCOA. **(F)** Bilateral PCOAs = P1 > 1 mm. **(G)** Arrow indicates he right slim P1. **(H)** Absent right P1.

The consistency of each circle of Willis diagnosis by two diagnostic physicians is displayed in Table 1. With DSA results as the standard, specificity, sensitivity, PPV, NPV and accuracy between VR, MIP, the original images, and the combined analyses are displayed in Table 2. Comparison of MRA and DSA for each circle of Willis type is shown in Figure 2.

**Table 1:** Consistency of the various circle of Willis types diagnosed by two observers (Kappa).

	**VR**	**MIP**	**Combined**
A	1	0.95	0.84
B	0.98	0.83	0.91
C	1	0.96	0.97
D	1	0.98	0.98
E	0.97	0.89	0.95
F	1	1	1
G	0.93	0.80	0.97
H	1	0.88	0.92

**Table 2:** Specificity, sensitivity, PPV, NPV, and accuracy between VR, MIP, the original images and the combined analysis of the various types the circle of Willis.

	**Sensitivity (%)**	**Specificity (%)**	**PPV(%)**	**NPV (%)**	**Accuracy(%)**
**VR**					
A	100	97.8	84.0	100	98.0
B	85.0	99.3	97.8	94.9	95.6
C	100	98.4	96.7	100	98.9
D	100	94.1	94.4	100	97.1
E	86.2	99.3	98.0	99.3	96.5
F	100	100	100	100	100
G	73.8	100	100	98.3	98.4
H	100	93.8	87.4	100	95.7
**MIP**					
A	100	98.3	87.5	100	98.5
B	88.3	99.6	98.7	96.2	96.8
C	100	98.7	97.2	100	99.1
D	100	96.1	96.2	100	98.0
E	89.2	99.6	98.8	96.1	96.8
F	100	100	100	100	100
G	81.8	100	100	99.0	99.0
H	100	95.5	90.5	100	96.9
**Com**					
A	100	100	100	100	100
B	95.8	99.7	99.1	98.7	98.8
C	100	100	100	100	100
D	100	98.0	98.0	100	99.0
E	96.1	99.9	99.6	98.7	98.9
F	100	100	100	100	100
G	90.0	100	100	99.5	99.5
H	100	98.3	95.9	100	98.7

The Kappa values of different types of Willis rings were observed in the range of 0.8–1 by different methods, suggesting a high degree of consistency between the the two observers.

Compared with VR and MIP, the sensitivity, specificity, PPV, NPV, and accuracy of combined analyses of each image was substantially better. The results agree well with DSA results.

Sensitivity and NPV of MRA images for A-, C-, D-, and H-types were all 100%, suggesting that MRA images are sensitive to the absence of blood vessels. Specificity, accuracy and sensitivity of the combined analyses for absence of ACOA were all 100%, consistent with DSA results.

Sensitivity, specificity, PPV, NPV and accuracy of MRA revealing F-type were all 100%, suggesting that MRA is sensitive and accurate for vessels with diameter ≥ 1 mm.

Sensitivity and PPV of VR images for the B-, E-, and G-types were relatively low (85.0, 86.2, and 73.8%, respectively). MIP was somewhat better (88.3, 89.2, and 81.8%, respectively). Combined analysis was better still (95.8, 96.1, and 99.0%, respectively). However, specificity and NPV were high (99.3–100%), suggesting that specificity of MRA revealing slim blood vessels was higher.

## Discussion

We demonstrated that high-resolution 3D-TOF-MRA compares well to DSA in evaluation of the structure of the circle of Willis. 3D-TOF-MRA is a non-invasive imaging method, which employs no contrast agent, no radiation, and does not require intubation of the patient. The method is convenient, cost-effective, and reproducible. 3D-TOF-MRA directly images vascular structures at multiple angles. These advantages suggest that the modality can serve as the preferred means of evaluating structural variations of the circle of Willis.

We used 3D-TOF-MRA with high resolution. We used a phased array coil and short TEs to increase the quality of MRA images. It was perfomed by using strong gradients and long acquisition times (Dagirmanjian et al., 1995). SENSE used parallel imaging to significantly shorten scan times and reduce artifacts while still maintaining good image quality. We used TONE to improve the display of outflow from a 3D volume. To smooth the uneven background suppression, which might be the main disadvantage of TONE, we simultaneously applied MOTSA and SLINKY (Liu and Rutt, 1998; Kirchhof et al., 2002). MOTSA useed TONE RF excitation to compensate for the saturation of the flow signal at the edge of the slab. Furthermore, SLINKY improved the display of vessels with slow blood flow, allowing visualization of complex vascular lesions.

This study demonstrates that original images consisting of VR and MIP were more sensitive and accurate than the single means for evaluating the structure of the circle of Willis. Because some loss of image information may occur during the process of reconstruction, the original images might provide more details, and the missed diagnosis rate might be reduced (Urchuk and Plewes, 1992). The original image can reveal not only thinner vessels, but also improved the accuracy of diagnosis by layer. In this study, the sensitivity, accuracy and specificity of the diagnosis were further improved by the combination of the original image and the reconstructed image. Because VR and MIP images can be observed by rotation, the overlapping of blood vessels can be greatly reduced. The diagnostic efficacy of the combination of the three approaches was close to that of DSA.

We did not perform the neck compression test with DSA, therefore the determination of A-type (ACOA absent) may be a certain deviation. In order to observe the opening of the ACOA, the contralateral carotid artery angiography was done by oppressing of the ipsilateral carotid artery. But the neck compression test will increase the duration and radiation dose, so it was not applicable to all patients. In the specific analysis of A-type in the DSA and MRA results, we found MRA positive rates as high as 85% of those diagnosed by DSA, while the other 15% may be related to the absence of the neck compression test. This suggests that the false negative rate of ACOA seen on MRA may be falsely elevated.

Sensitivity, specificity, PPV, NPV, and accuracy of F-type diagnosis by MRA were 100%, suggesting that MRA is sensitive and accurate for vessels with diameter ≥1 mm. The diagnostic performance was equivalent to DSA. In addition, we showed that sensitivity and PPV of VR images for the B-, E-, and G-type were relatively low, MIP was somewhat better, and combined analyses were better still. However, the specificity and NPV of the three methods were high, suggesting that MRA is not sensitive enough to demonstrate slim blood vessels, but is nevertheless highly accurate.

Previous studies have shown that the overall sensitivity of MRA is >80%, proving the presence or absence of arterial segments of the circle of Willis (Fürst et al., 1993; Patrux et al., 1994; Stock et al., 1996; Hendrikse et al., 2008a). Specificity of MRA (63–100%) varied depending on the MRA technology and analysis methods. Stock et al. (1996) performed MRA on a 1.5T system. They found that MIP images had a sensitivity of 87% and a specificity of 88%. The MRA source images depicted a vascular segment with a sensitivity of 89% and a specificity of 63%. The PPV of an arterial segment with a diameter of at least 1 mm was 99%. Hendrikse et al. (2008a) compared 3D-TOF-MRA with 2D phase contrast images with DSA. They found that the sensitivity and specificity of collateral blood flow measurements at the anterior portion of the Willis ring were 83 and 77%, respectively. However, the sensitivity of MRA to PCOA is low (33%). In this study, VR showed PCOA sensitivity of 86.2%, MIP of 89.2%, with combined analysis sensitivity increasing to 96.1%. Our study not only had a larger sample size than the earlier published articles, but also the MRA examinations were performed on a 3T system whose images return higher resolution. The sensitivity, specificity and sensitivity were substantially improved, especially combined analysis: 90–100, 98–100%, respectively. PPV for arterial segments at least 1 mm in diameter was 100%.

A CTA study of the circle of Willis reported sensitivity and specificity >90%. However, the description of structural dysplasia had certain limitations. Sensitivity was 52.6%, specificity was 98.3% (Han et al., 2011). But there are well-known disadvantages, such as radiation exposure, renal toxicity due to contrast agent, and adverse effects on bone ossification and calcification. All this points to the superiority of MRA.

The use of 3D-TOF-MRA technology provides the means to perform large-scale epidemiological surveys, and to establish a morphological quantitative imaging database of the circle of Willis and the basilar artery branches. There the modality could contribute to understanding the pathogenesis, diagnosis, and treatment of cerebrovascular disease.

## Data Availability Statement

All datasets generated for this study are included in the article/supplementary material.

## Ethics Statement

The studies involving human participants were reviewed and approved by Ethics committee of the Shanghai sixth people's hospital affiliated to Shanghai Jiao Tong university. The patients/participants provided their written informed consent to participate in this study.

## Author Contributions

Y-HL and Y-WZ contributed conception and design of the study. X-EW organized the database. FW performed the statistical analysis. JL wrote the first draft of the manuscript. JW wrote sections of the manuscript. All authors contributed to manuscript revision, read, and approved the submitted version.

### Conflict of Interest

The authors declare that the research was conducted in the absence of any commercial or financial relationships that could be construed as a potential conflict of interest.
